# Nicaraven Attenuates Postoperative Systemic Inflammatory Responses-Induced Tumor Metastasis

**DOI:** 10.1245/s10434-019-08076-2

**Published:** 2019-12-23

**Authors:** Xu Zhang, Takahito Moriwaki, Tsuyoshi Kawabata, Shinji Goto, Ke-Xiang Liu, Chang-Ying Guo, Tao-Sheng Li

**Affiliations:** 1 Department of Stem Cell Biology, Nagasaki University Graduate School of Biomedical Sciences, 1-12-4 Sakamoto, Nagasaki, 852-8523 Japan; 2 Department of Cardiovascular Surgery, The Second Hospital of Jilin University, Ziqiang Street 218, Changchun, Jilin 130041 China; 3 Department of Thoracic Surgery, Jiangxi Cancer Hospital, No. 519 Beijing East Road, Nanchang, 330006 China

## Abstract

**Background:**

Inflammation has been demonstrated to promote cancer metastasis. Due to the well-known systemic inflammatory responses (SIR) after major surgery, it is critical to investigate and attenuate SIR-induced tumor metastasis of cancer patients suffering surgical procedures.

**Methods:**

C57BL/6 mice were intravenously injected with Lewis lung cancer cells at 6, 24, and 72 h after the induction of intestinal ischemia/reperfusion (I/R) injury. We found that the number of tumor nodules significantly increased in lungs of mice injected with cancer cells at 6 h but not at 24 and 72 h after I/R injury. The administration of nicaraven 30 min before and 24 h after I/R injury effectively attenuated the enhanced tumor metastasis to lungs. Protein array showed the increase of various cytokines in plasma of mice at 6 h after I/R injury, but many of them were attenuated by the administration of nicaraven. Immunostaining indicated the increase of Ly6g-, CD206-, and CD11c-positive inflammatory cells in the lungs, but it was also attenuated by nicaraven administration.

**Conclusions:**

Postoperative SIR-induced tumor metastasis have been clearly evidenced in our experimental model, and the administration of nicaraven may ameliorate the SIR-induced tumor metastasis by suppressing inflammatory responses.

Metastatic development is the most common causes of death for cancer patients.^1^,^2^ It has recently been demonstrated that excessive production of cytokines/chemokines and the increased infiltration of inflammatory cells promote cancer metastasis to the lungs.^3^

Beyond bacterial/viral infections, systemic inflammatory responses (SIR) can be widely caused by various pathological conditions, such as major surgery and other noninfectious severe damages/injuries of tissues/organs. Because surgical resection is one of the most effective treatments for cancer and some cancer patients also may suffer to surgical procedures due to other severe diseases, it is critical to investigate whether extensive surgical procedures promote cancer metastasis. If so, it also is highly required to find potential intervention treatment.

Nicaraven, a powerful free radical scavenger, has been demonstrated to effectively protect against ischemia–reperfusion (I/R) injury of various tissues/organs.^4^^–^6 We have recently further demonstrated that nicaraven attenuates the enhanced tumor metastasis to lungs after thoracic radiation exposure, which likely associates with the decreased cytokines/chemokines in plasma and the infiltration of inflammatory cells in lungs.^3^ Taking the anti-inflammatory role of nicaraven into consideration, it is quite possible that nicaraven also inhibits the SIR-induced cancer metastasis after major surgery.

Using an intestinal I/R injury model in mice, we investigated the postoperative SIR-induced tumor metastasis. Our data have confirmed the postoperative SIR-induced lung metastasis, which effectively ameliorated by temporarily administration with nicaraven.

## Materials and Methods

### Cell Culture

Mouse Lewis lung carcinoma (LLC) cells used for the experiments were maintained in Dulbecco’s modified Eagle’s medium (DMEM) (Wako, Japan) supplemented with 10% fetal bovine serum and 1% penicillin/streptomycin (Gibco, USA) at 37 °C in a humidified incubator under 5% CO_2_ and 95% air.

### Experimental Animals

C57BL/6 male mice (CLEA, Japan), 9 to 12 weeks old, were used in this study. The animals were bred in specific, pathogen-free conditions and were allowed free access to food and water in a temperature-controlled environment with a 12:12-h light–dark cycle. All experiments were approved by the Institutional Animal Care and Use Committee of Nagasaki University (No. 1608251335-9), and animal procedures were performed in accordance with institutional and national guidelines. At the end of the experiments, mice were administered general anesthesia by an intraperitoneal injection of mixed anesthetic (0.75 mg/kg medetomidine, 4 mg/kg midazolam, 5 mg/kg butorphanol) and euthanized by severing the aorta.

### Intestinal I/R Injury and Experimental Lung Cancer Metastasis Models

Intestinal I/R injury model was established as previously described.^7^,^8^ Briefly, the mice were deprived of food and water for 8 h before experimental procedures in which mice were implemented under a heating lamp to preserve the body temperature. Mice were received laparotomy, a 1.5-cm medial abdomen incision, under general anesthesia. The terminal collateral branches of superior mesenteric artery were ligated for 45 min using several non-traumatic microvascular clamps and then followed by reperfusion (*n* = 12, IR group). Healthy mice received laparotomy only were used as the control (*n* = 12, LP group).

Experimental lung cancer metastasis were induced to mice by intravenous injection with LLC cells (5 × 10^5^ cells in 0.5 ml of saline) at 6, 24, and 72 h after surgical procedures. Animals were sacrificed 5 weeks later, and lung tissues were excised and weighed. We also counted tumor nodules on the surface of lungs.

### Nicaraven Administration

To investigate whether nicaraven attenuates intestinal I/R injury-induced cancer metastasis, mice were intravenously injected with LLC cells at 6 h after intestinal I/R injury, and then randomly received intraperitoneal injection with 100 mg/kg of nicaraven (*n* = 6, IR + N group) or saline (*n* = 6, IR group) 30 min before and 24 h after surgical procedures. For another control, mice were injected with LLC cells at 6 h after laparotomy and then given saline injections (*n* = 6, LP group). All animals were sacrificed 5 weeks later, and lung tissues were excised and weighed. We counted tumor nodules on the surface of lungs.

### Proteome Profiler™ Array

To explore the mechanism, mice were randomly received intraperitoneal injection with 100 mg/kg of nicaraven (*n* = 3, IR + N group) or saline (*n* = 3, IR group) 30 min before and immediately after intestinal I/R injury. Mice received laparotomy and saline injection were also used for control (*n* = 3, LP group). We killed the mice and collected the plasma and lung tissues (for immunofluorescence stainings) at 6 h after treatments. We mixed the plasma from three mice of each group, and the average levels of cytokines in plasma were compared among groups using mouse cytokine Proteome Profiler™ array (ARY006, R&D Systems) per manufacturer’s instructions. Briefly, premixed plasma/antibody cocktails were incubated with blocked membranes overnight at 4 °C on a rocking platform shaker. After three washes, membranes were incubated with diluted streptavidin-HRP for 30 min and washed three times. Images of the blots were acquired, and semiquantitative analyses of cytokine or chemokine spots were performed using an Imagequant™ LAS-4000 mini biomolecular imager (GE Healthcare Bio-Sciences).

### Immunofluorescence Stainings

To detect the infiltration of inflammatory cells, lung tissues embedded in Tissue-Tek^®^ O.C.T. compound were cut into 8-µm-thick sections for immunofluorescence staining. Briefly, frozen sections were fixed with 4% formalin (Wako) for 10 min at 4 °C. After blocking, tissue sections were incubated with rat anti-mouse Ly6 g antibody (1:100 dilution, Abcam), goat anti-mouse CD206 antibody (1:100 dilution, R&D Systems), and mouse anti-mouse CD11c antibody (1:150 dilution, Abcam) overnight at 4 °C. After washing, tissue sections were incubated with Alexa Fluorescent 594-conjugated anti-rat Ig (1:400 dilution), Alexa Fluorescent 488-conjugated anti-goat Ig (1:200 dilution), and Alexa Fluorescent 546 anti-mouse Ig (1:400 dilution) secondary antibodies, respectively, at room temperature for 1 h in the dark. The immunofluorescence was examined under microscope (Olympus).

### Statistical Analysis

All the values were presented as the mean ± SD. Statistical significance was determined by one-way analysis of variance (ANOVA) followed by Tukey’s test (Dr. SPSS II, Chicago, IL). A *p* value < 0.05 was accepted as significant.

## Results

### Intestinal I/R Injury Significantly Promoted Tumor Metastasis to Lungs

**Fig. 1:**
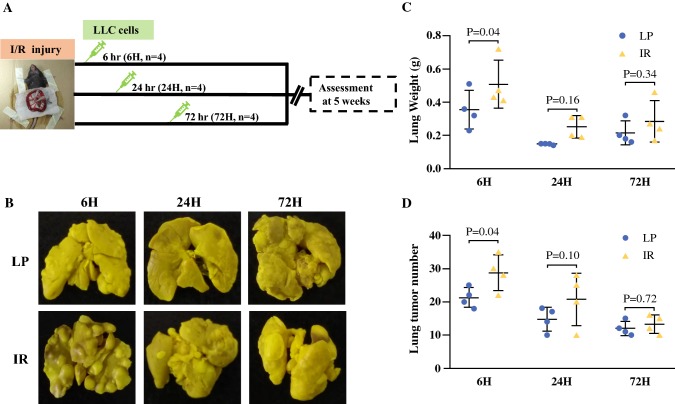
Lung metastasis in mice received intravenous injection with Lewis lung cancer cells at 6, 24 and 72 h after intestinal I/R injury (IR) or laparotomy (LP). **a** Schematic diagram about the timeline of the experimental protocol. **b** Representative images show the tumor nodules in lungs. Quantitative data on the weight of whole lung tissues (**c**) and the numbers of tumor nodules in lungs (**d**). Data are presented as the mean ± SD, *n* = 4 in each group

As shown in the schematic timeline of Fig. 1a about the experimental protocol, we intravenously injected with LLC cells to mice at 6, 24, and 72 h after intestinal I/R injury or sham laparotomy and examined tumor metastasis in lungs 5 weeks later. Compared with sham laparotomy, our data showed that the number of tumor nodules on the surfaces of lungs was significantly higher in mice only when we injected the LLC cells at 6 h but not at 24 and 72 h after intestinal I/R injury (Fig. 1b, c).

### Nicaraven Administration Attenuated Lung Metastasis Induced by Intestinal I/R Injury

**Fig. 2:**
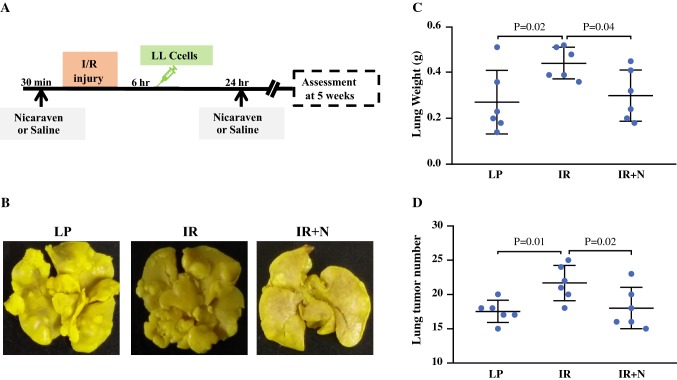
The effect of nicaraven on attenuating lung metastasis induced by intestinal I/R injury. **a** Schematic diagram about the timeline of the experimental protocol. **b** Representative images show the tumor nodules in lungs. Quantitative data on the weight of whole lung tissues (**c**) and the numbers of tumor nodules in lungs (**d**). Data are presented as the mean ± SD, *n* = 6 in each group. *LP* laparotomy; *IR* ischemia reperfusion; *IR *+ *N* ischemia reperfusion with nicaraven treatment

To investigate whether nicaraven effectively attenuates the intestinal I/R injury-induced lung metastasis, we intraperitoneally injected nicaraven to mice 30 min before and 24 h after intestinal I/R injury (Fig. 2a). As expected, the number of tumor nodules in lungs were significantly increased if we injected the LLC cells to mice at 6 h after intestinal I/R injury, but the enhanced tumor metastasis was almost attenuated by nicaraven administration (Fig. 2b–d).

### Nicaraven Administration Inhibited the Systemic Inflammatory Cytokines in Mice After Intestinal I/R Injury

**Fig. 3:**
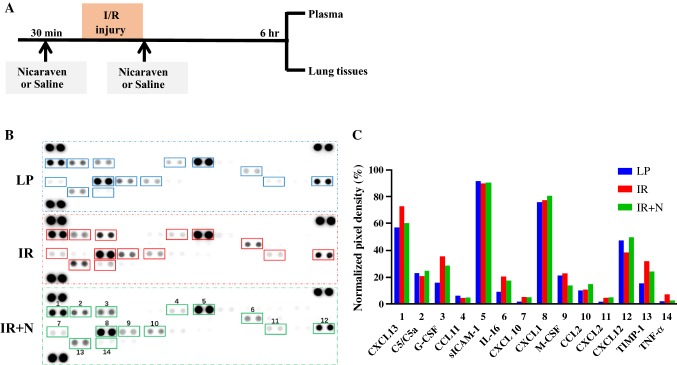
Detection of inflammatory cytokines in plasma by Proteome Profiler™ array. **a** Schematic diagram about the timeline of the experimental protocol. **b** Representative images on the expression of 40 cytokines/chemokines in plasma of mice received laparotomy and saline injections (LP), intestinal I/R injury and saline injections (IR), or intestinal I/R injury and nicaraven administration (IR + N). **c** Bar graph shows the semiquantitative data about the indicated cytokines/chemokines. Data represent as normalized pixel density

To explore the mechanism, we given nicaraven to mice 30 min before and soon after I/R injury and evaluated the average levels of inflammatory cytokines in plasma of three mice from each group by proteome Profiler™ array at 6 h after treatments (Fig. 3a). Compared with the mice received laparotomy, the average levels of various inflammatory cytokines in plasma, such as CXCL13, G-CSF, M-CSF, IL-16, TIMP-1, and TNF-α, were detected higher in the mice suffered to intestinal I/R injury (Fig. 3b, c), suggesting the systemic inflammatory responses after intestinal I/R injury. However, nicaraven administration almost inhibited the release of these inflammatory cytokines induced by intestinal I/R injury (Fig. 3b, c).

### Nicaraven Administration Reduced the Inflammatory Cells in Lung Tissues of Mice aFter Intestinal I/R Injury

**Fig. 4:**
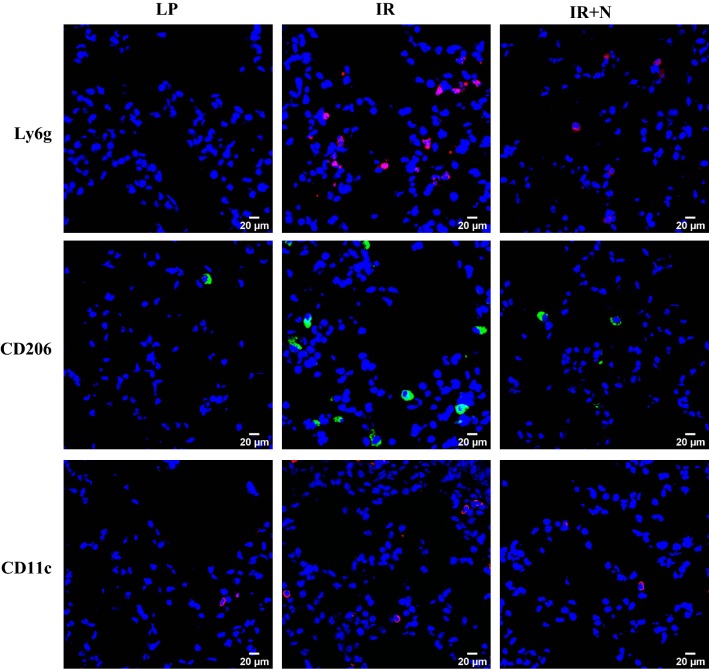
The infiltration of inflammatory cells in lung tissues. Representative images of immunofluorescence staining show the Ly6g-, CD206-, and CD11c-positive inflammatory cells within lung tissues. Nuclei were counterstained with 4′,6-diamidino-2-phenylindole (DAPI, blue). *LP* laparotomy; *IR* ischemia reperfusion; *IR *+ *N* IR with nicaraven administration

Immunofluorescence staining was performed to detect the infiltration of inflammatory cells in lung tissues at 6 h after treatments. As shown in the representative images (Fig. 4), the Ly6g-, CD206-, and CD11c-positive inflammatory cells were highlighted within lung tissues of mice suffered to intestinal I/R injury in comparison with mice received laparotomy only. However, the infiltration of Ly6g-, CD206-, and CD11c-positive inflammatory cells was obviously reduced by nicaraven administration.

## Discussion

Extensive surgical procedures are generally known to cause SIR with an increased systemic inflammatory cytokines.^9^ As a hyperinflammatory microenvironment has been demonstrated to promote tumor metastasis, we investigated whether major surgical procedures also promoted tumor metastasis.^10^^–^13 Recently, we have found that nicaraven, a hydroxyl radical-specific scavenger, effectively protects normal tissue cells against radiation-induced injuries by suppressing the expression of inflammatory cytokines/chemokines.^3^,^14^ Therefore, we also tested whether nicaraven could attenuate postoperative SIR-induced tumor metastasis.

As expected, many cytokines/chemokines were obviously increased in plasma of mice at 6 h after intestinal I/R injury, suggesting systemic inflammatory responses. Using an experimental metastasis model, the number of tumor nodules were increased in lungs when the cancer cells were injected into the mice at 6 h but not at 24 and 72 h after intestinal I/R injury. Although we did not investigate the kinetics of cytokines/chemokines at systemic and regional levels, a particular time window on postoperative SIR-induced tumor metastasis might be associated with the dynamic changes of inflammatory responses following intestinal I/R injury.

Agreed well with the results of our previous studies,^3^,^15^ the administration of nicaraven to mice 30 min before and immediately after intestinal I/R injury decreased the levels of some cytokines/chemokines, such as TNF-α and CXCL1 in plasma and reduced the infiltration of Ly6 g^+^ neutrophils and CD206^+^/CD11c^+^ macrophages in lungs. Moreover, the enhanced tumor metastasis in lungs of mice suffered to intestinal I/R injury was almost completely attenuated by nicaraven administration. The timing and dosage of nicaraven administration were based on our past studies, but it was required to be further validated by additional experiments.^3^,^10^,^15^

Increasing evidences have shown the relationship between inflammatory cytokines/cells and tumor metastasis in recent years. It has been reported that TNF-α enhances tumor invasiveness and alters the tumor microenvironment to promote metastasis.^16^ CXCL1/CXCR2 signaling pathway is also known to regulate tumor growth and promote tumor metastasis.^17^,^18^ Recent study has further demonstrated that Ly6g^+^ neutrophils specifically support metastatic initiation in premetastatic site.^12^ The CD206^+^/CD11c^+^ macrophages have been classified as protumoral and proinflammatory macrophages.^19^,^20^ Although the lack of direct evidence about the causal relationship between tumor metastasis and inflammatory responses following I/R injury in the present study, it is reasonable to speculate that nicaraven effectively attenuates postoperative SIR-induced tumor metastasis through the inhibition of inflammatory responses.

This study had some limitations. First, we did not measure the circulating neutrophils/lymphocytes and the levels of cytokines/chemokines in lung tissues. Therefore, it is unclear about the detailed mechanism on the increased infiltration of inflammatory cells in lungs of mice suffered to intestinal I/R injury. Second, we did not verify our finding by further interventional experiments, such as the blockade of specific cytokines/chemokines and signaling pathways, which will help us to identify the key cytokines/chemokines and inflammatory cells in promoting tumor metastasis. Otherwise, we did not evaluate the morphological and functional changes in endothelial cells, which is known to also play critical role in experimental tumor metastasis.

## Conclusions

Our experimental data in an intestinal I/R injury model confirmed postoperative SIR-induced tumor metastasis at an acute phase. Temporary administration of nicaraven effectively inhibited inflammatory responses and attenuated postoperative SIR-induced tumor metastasis. Considering the well-documented safety profile and anti-inflammatory effect, nicaraven may be a potentially effective agent for cancer patients who suffered to surgical procedures.^4^
